# Combining Big Data and Artificial Intelligence for Managing Collective Knowledge in Unpredictable Environment—Insights from the Chinese Case in Facing COVID-19

**DOI:** 10.1007/s13132-020-00703-8

**Published:** 2020-11-13

**Authors:** Francesca Iandolo, Francesca Loia, Irene Fulco, Chiara Nespoli, Francesco Caputo

**Affiliations:** 1 Department of Management, Faculty of Economics, University of Rome ‘La Sapienza’, Via del Castro Laurenziano, 9, 00161 Rome, Italy; 2 Department of Economics, Management, and Institutions (DEMI), University of Naples ‘Federico II’, Via Cintia, 21, 80126 Naples, Italy; 3 Department of Economics and Management (DEIM), University of Tuscia of Viterbo, Via del Paradiso, 47, 01100 Viterbo, Italy; 4 University of Campania ‘L. Vanvitelli’, Via Roma, 29, 81031 Aversa, CE Italy

**Keywords:** Knowledge management, Collective knowledge, Big data, Artificial intelligence, Viable systems approach, COVID-19

## Abstract

The increasing fluidity of social and business configurations made possible by the opportunities provided by the World Wide Web and the new technologies is questioning the validity of consolidated business models and managerial approaches. New rules are emerging and multiple changes are required to both individuals and organizations engaged in dynamic and unpredictable paths.

In such a scenario, the paper aims at describing the potential role of big data and artificial intelligence in the path toward a collective approach to knowledge management. Thanks to the interpretative lens provided by systems thinking, a framework able to explain human-machine interaction is depicted and its contribution to the definition of a collective approach to knowledge management in unpredictable environment is traced.

Reflections herein are briefly discussed with reference to the Chinese governmental approach for managing COVID-19 spread to emphasise the support that a technology-based collective approach to knowledge management can provide to decision-making processes in unpredictable environments.

## Preliminary Reflections About the Contribution of New Technologies to Collective Approach for Knowledge Management

The increasing rapidity of social and business changes is rapidly producing several consequences on consolidated business and managerial models (Kirton 2004; Van Oosterhout et al. 2006; Zainon et al. 2011; Bourdieu et al. 2019). Previous approaches are showing increasing incapability in forecasting emerging dynamics pushing both researchers and practitioners toward new possible models and technical instruments.

Social and business organizations are experiencing a new era in which competitive advantages should be daily rebuilt and they cannot be more managed by individual actors (Liedtka 1996; Ketchen Jr et al. 2007; Allred et al. 2011). New rules are emerging in which market and social balances daily change as a consequence of interactions among multiple categories of actors endowed by subjective purposes and—sometimes—conflicting approaches (Del Giudice et al. 2016).

Organizations interested in surviving over the time must rethink their strategies and managerial paths overcoming a ‘simplistic’ view based on previous consolidated market position and/or contributions provided by the efficiency in production (Sweet 2001; Sandberg 2014). Something more is required for all the organizations involved in the emerging era (Kanter 2003; Saviano et al. 2017). New processes should be built acting on the collaboration and contamination among divergent perspectives to overcome reductionist boundary-based representations for identifying new business areas and market opportunities (Caputo 2016; Del Giudice et al. 2017; Arrigo 2018).

Collaboration, contamination, interactions, and sharing among the others seem to be new keywords on which to base a paradigmatic shift for business and managerial studies (Saviano et al. 2018; Yu and Yang 2018).

All these concepts are well summarized by the domain of the Digital Era as an emerging time-based configuration in which relationships among the actors are influenced by individual ability to be actively involved in emerging technology-based configurations and competitive advantages emerge from the combination of individual abilities thanks to the contributions provided by new technologies (Christensen 2001; Hu et al. 2005; Caputo and Walletzky 2017; Amendola et al. 2018). Digital era offers to all social and business actors the possibility for becoming central nodes in emerging and changing organizations within rules are dynamically defined as a consequence of the total amount of data shared among the actors and knowledge co-produced by the interactions among the parts (Tapscott 1996; Brynjolfsson and Kahin 2002; Turban et al. 2006). Accordingly, digital era can be considered as a technology-based configuration of knowledge era in which centrality of data and information is ensured while new ‘weapons’ are provided to all the actors interested in better understanding and managing dynamics and emerging processes (Leeflang et al. 2014).

Recognizing the multiple challenges that can derive from above-mentioned configurations for all the organizations, the paper aims at reflecting about the influence of new technologies in knowledge management and decision-making processes to identify possible ways through which consolidated and almost inefficient individualistic approaches can be outmoded for identifying and catching new market opportunities. In such a direction, the interpretative framework of viable systems approach (VSA) (Barile 2009; Golinelli 2010; Barile et al. 2012) is embraced for providing a wider view about paths through which a collective approach to knowledge management can be stimulated for better understanding and solving shared problems. In such a direction, knowledge management domain in investigated with the aim to explore dynamics on which big data and artificial intelligence are based and through which collective knowledge can emerge and support the management of unpredictable environment. Policy-making approaches developed in China for facing COVID-19 challenge are then briefly discussed for underlining the multiple advantages that a collective approach to knowledge management can produce both for individuals and organizations in the management of unpredictable environments. The attention is focused on Chinese case in facing COVID-19 because it well summarizes both individual and collective reaction to the emerging and unpredictable dynamics. At the same time, the observed case also provides multiple interesting evidences about the role of new technologies in supporting knowledge contamination for facing collective issues.

The remainder of this paper is structured as follows: “Theoretical background” provides a brief overview about the theoretical background on which reflections herein are based with specific reference to the role of new technologies in the knowledge management process and to the challenges and opportunities of collective approaches in knowledge management; “A viable systems perspective for managing knowledge in the digital era” introduces the conceptual framework of VSA and it underlines possible systems-based guidelines for supporting collective approaches to knowledge management in the digital era; “Collective intelligence for facing COVID-19 challenge: the Chinese case” provides a synthesis of policy-making devolved in China for facing COVID-19 challenge to emphasise implications and opportunities of a systems-based view of collective approaches to knowledge management process; “Implications, limitations, and future directions for research” finally lists main theoretical and practical implications of reflections herein and it also draws main conclusions and directions for future research.

## Theoretical Background

### Insights from Digital Tools in Knowledge Management Processes

Nowadays, information technologies made cheaper and faster gathering and processing of large amounts of data (Asrar-ul-Haq and Anwar 2016; Santoro et al. 2018). This new phenomenon, called big data, represents a new era in data exploration and usage (Chen and Zhang 2014). The “*mass digitization*” (Coyle 2006) along with “Internet of Things” interconnection (Ashton 2009) has led to a rapid expansion of large amounts of data along three dimensions: *volume*, *speed*, and *variety*, as summarized by the “3Vs” model of Laney (Zikopoulos and Eaton 2011; Beyer and Laney 2012; Zaslavsky et al. 2013). Over the time, additional dimensions have been added to this model for highlighting the quality across datasets—*veracity*—and the capacity to generate useful output for industry challenges and issues—*value* (Uddin and Gupta 2014).

Big data analytics follow an approach based on artificial intelligence (AI), which can be defined as the way of training computers to mimic thinking patterns (Nilsson 2014). The subfields of AI include several techniques of machine learning (Qiu et al. 2016; Zhou et al. 2017) through which it is possible to build a computer system able to ‘change’ thanks to experience. This approach offers the opportunity for identifying non-obvious and hidden patterns of information and building predictive models.

In such a direction, big data can be considered as an additional valuable knowledge asset (Erickson and Rothberg 2015): the huge volumes of data are processed for extracting valuable knowledge, which can be used for enhancing the performance of many different processes in organizations.

The adoption of big data instruments in organizations’ configurations determines the capacity to absorb untapped knowledge and combine it in innovative paths for improving organizations’ performance. As a valuable example, the combination of tacit knowledge of experienced employees (Ball and Gotsill 2011) with the knowledge obtained from big data (Feblowitz 2013) offers to organizations valuable new resources on which found new competitive advantages.

Thanks to big data, organizations can collect information from different perspectives with the aim to define more efficient decision-making processes (Lamont 2012) based on a more detailed understanding of existing data (LaValle et al. 2011) and on the opportunities for better tracing further social and market evolutions (Bose 2009; Pauleen and Wang 2017).

Accordingly, big data can support decision-making process improving efficiency in data’ transmission, capture, storage, analysis, visualization, and interpretation (LaValle et al. 2010; Chen and Zhang 2014). More in detail, the algorithm-based approaches offer the possibility for extracting useful information from large datasets (Fan and Bifet 2013; Daňa et al. 2018) and it can reveal valuable insights on which to base decision-making process for overcoming limitations related to reductionist approaches only based on experience and intuition (McAfee et al. 2012).

### A Collective Approach to Knowledge Management

As a consequence of changing dynamics in social and business configurations, organizations interested in surviving over the time must develop frameworks able to conceptually and operationally integrate smart technologies in knowledge management processes for supporting decision-making and organizational value creation activities (Tanriverdi 2005; Holtshouse 2013; Carayannis et al. 2018).

Accordingly, Gruber (2008) reflecting upon web 2.0 era, characterized by blogs, Twitter, wikis, photo sharing, collaborative tagging and social networking sites, which enable to create and disseminate contents in a relatively simple way, has proposed collective knowledge system as a framework able to reveal the power of social web thanks to the semantic web.

Following Gruber’s representation (2008), social web refers to web sites in which user participation is the primary value driver, for instance, Facebook and YouTube, while semantic web can be considered as an ecosystem of structured data in which value is created by data integration from many sources. More in detail, semantic web aims at providing answers to small groups of people by elaborating user-generated contents through knowledge extraction approaches (Hepp et al. 2005) supporting the shift from gathered and individual intelligence to collective intelligence (Davies et al. 2003; Handschuh and Staab 2003).

**Fig. 1:**
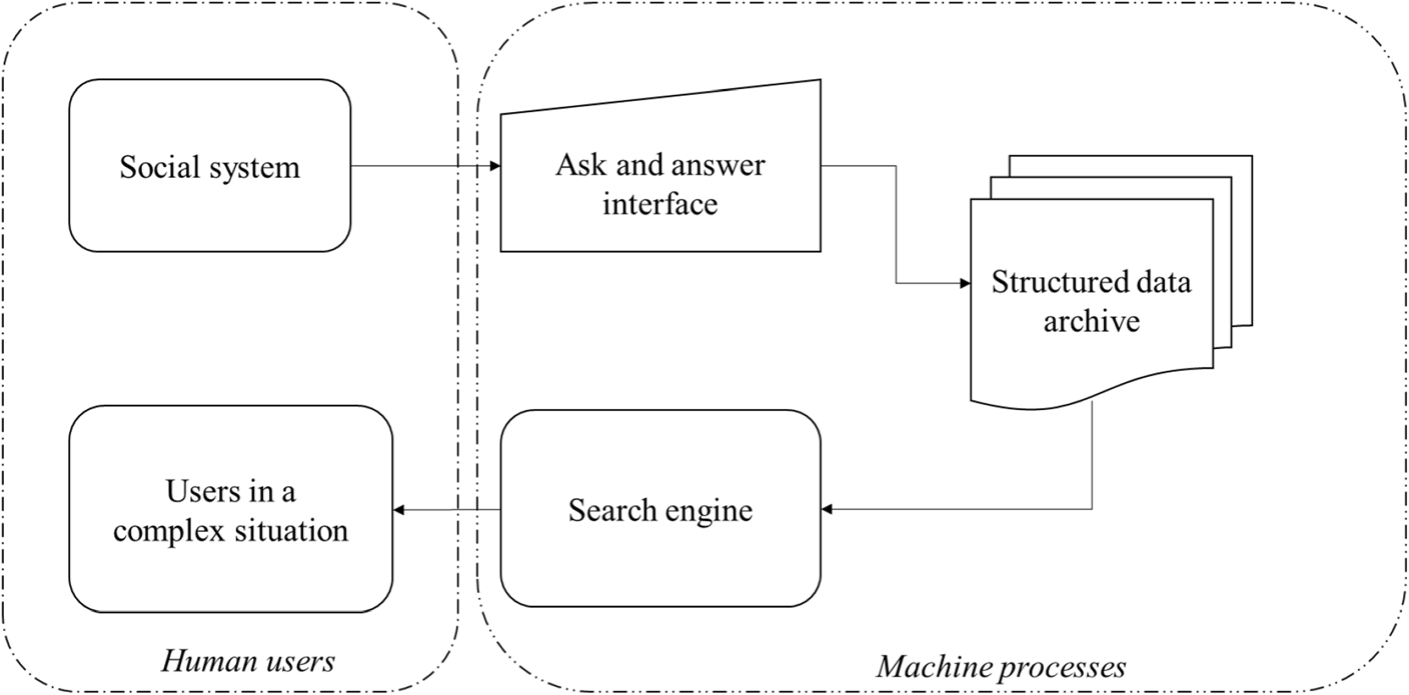
Collective knowledge system. Source: Authors’ elaboration from Gruber (2008)

Collective intelligence can be considered as the result of an emerging environment in which social knowledge is enhanced thanks to the support provided by networked ICTs (information communication technologies) as drivers able to simultaneously and constantly enforce human interactions (Lévy and Bononno 1997). As widely discussed by consolidate literature, collective intelligence has a pervasive and disruptive role in defining human-machine relationship (de Senzi Zancul et al. 2016; Khan and Vorley 2017; Soto-Acosta et al. 2018). In such a direction, collective intelligence offers the opportunity for defining new schemes in knowledge management processes thanks to the definition of a collective knowledge system (CKS) as a framework structured in three levels as depicted in following Fig. 1:

- A *social system* related to problem-solving discussions (questions and answers) developed in websites;
- A *search engine* able to identify and classify contents generated in the social system;
- *Users in a complex situation* related to users’ actions interested in obtaining information through the definition of queries or in providing information through feedback processes related to the efficiency and utility of existing queries and search mechanisms.

As shown in Fig. 1, CKS refers to a human-machine interaction framework in which both humans and machines actively contribute to the production and usage of intelligence useful for facing emerging problems thanks to the supports provided by ICT tools (analytics and research engine, etc.) (Gaeta et al. 2019). Following this flow, knowledge emerges as the result of combination among individual contributions and their reciprocal influence (Pauleen and Wang 2017; Sumbal et al. 2017).

In the field of emergency response, a framework based on the collective intelligence can aggregate multiple sources of knowledge reducing time for data processing and supporting decision-making processes (Vivacqua and Borges 2010). In such a scenario, Vivacqua and Borges (2012) define a framework which formalizes the interaction between environmental information (*context of the emergency*) and the multiple involved individuals (*population and respondents*) with the aim to trace paths through which individuals can provide information for supporting decision-making and policy-making processes. Basically, the framework is composed of two modules:

- A Collective Intelligence (CI) module composed by:
- An *interface* trough which population can provide data about their perceptions;
- A *data collection* system for receiving and store raw data sent in by the individuals;
- A *data processing* system in which multiple algorithms co-exist for aggregating, organizing, classifying, consolidating, and verifying collected data.
- A *Decision Support module* that receives CI data, processes it, integrates it with data from external sources, and provides information useful for supporting decision-making processes.

Relevant advantages of CKS in managing emergent and unpredictable dynamics are also highlighted by the *black swan* metaphor proposed by Taleb (2007), in which it underlined the need for connecting relevant information from individuals and groups in order to manage not expected events.

In nutshell, a collective knowledge framework can support knowledge management system for preventing black swans. However, there is still an open question about the ways in which collective knowledge frameworks can be effectively built and spread (Hecker 2012). For bridging this gap in knowledge, useful guidelines can be derived from systems thinking as summarized in the following section.

## A Viable Systems Perspective for Managing Knowledge in the Digital Era

VSA is a conceptual framework based on systems theories (Beer 1984; Capra 1997; Checkland 1981; Laszlo 1996; Meadows 2008; Von Bertalanffy 1968; Weinberg 1975) which provide useful guidelines for supporting both researchers and practitioners in analysing, understanding, and systems behaviours. According to VSA (Barile 2009; Golinelli 2010), each organized entity that is interested in survival over time can be described as a viable system. Focusing on the relationship between environment and system (Ashby 1968), the VSA states that a viable system is able to achieve balancing conditions and to survive if it is able to understand and respond to the complexity and turbulence of the external environment. Accordingly, the VSA highlights a strong link between decision-making processes and systems’ survival, clarifying that decision-making is a cognitive process within multiple phases should be managed as summarized in following Table 1.

**Table 1:** The knowledge itinerary

Phase of the decision-making process	Brief description
Chaos	Conditions in which it is perceived the existence of ‘something wrong or strange’ but origin/cause, effect, and solution of this condition are not known.
Complexity	Problems are understood and it appears as clear in the system’s perspective, but any solution is imaged and/or known.
Complication	Systems perceive the existence of a possible solution for the experienced problem, but it is still not well defined and/or formalized.
Certainty	Perceived solutions have been applied and problems have been solved then the system understands that it is possible to solve similar problems using the same instruments and resolutive approaches.

Source: Authors’ elaboration from Barile (2009)

Each phase summarized in Fig. 1 requires a different ‘kind of knowledge’ in terms of combination among (Barile 2013): (1) *information units* as the amount of data endowed by a system; (2) *interpretative schemes* as organizational patterns through which data are organized; and (3) *value category* as values and strong beliefs that address system’s evaluation and perceptions.

Recognizing the interpretative contributions provided by the VSA, it is possible to state that acting on the paths through which information units, interpretative schemes, and value categories interact both inside a system and among multiple systems, different decision-making processes can be derived. Building upon this speculation, a renovate role for big data and AI can be identified with reference to emergent and chaotic configurations.

A human-machine-based interactions model can be formulated acting on the interaction among two levels (Gruber 2008):

- *Humans users* which include the environmental dynamics and human-human interactions;
- *Machine processes* related to automated activities carried out by software and hardware components for enabling the smart technologies processes. This level is composed by the *Collective Intelligence (CI) module*, which aims to collect and process the user-generated data, and the *Decision Support module* that elaborates through AI algorithms the information units gathered.

**Fig. 2:**
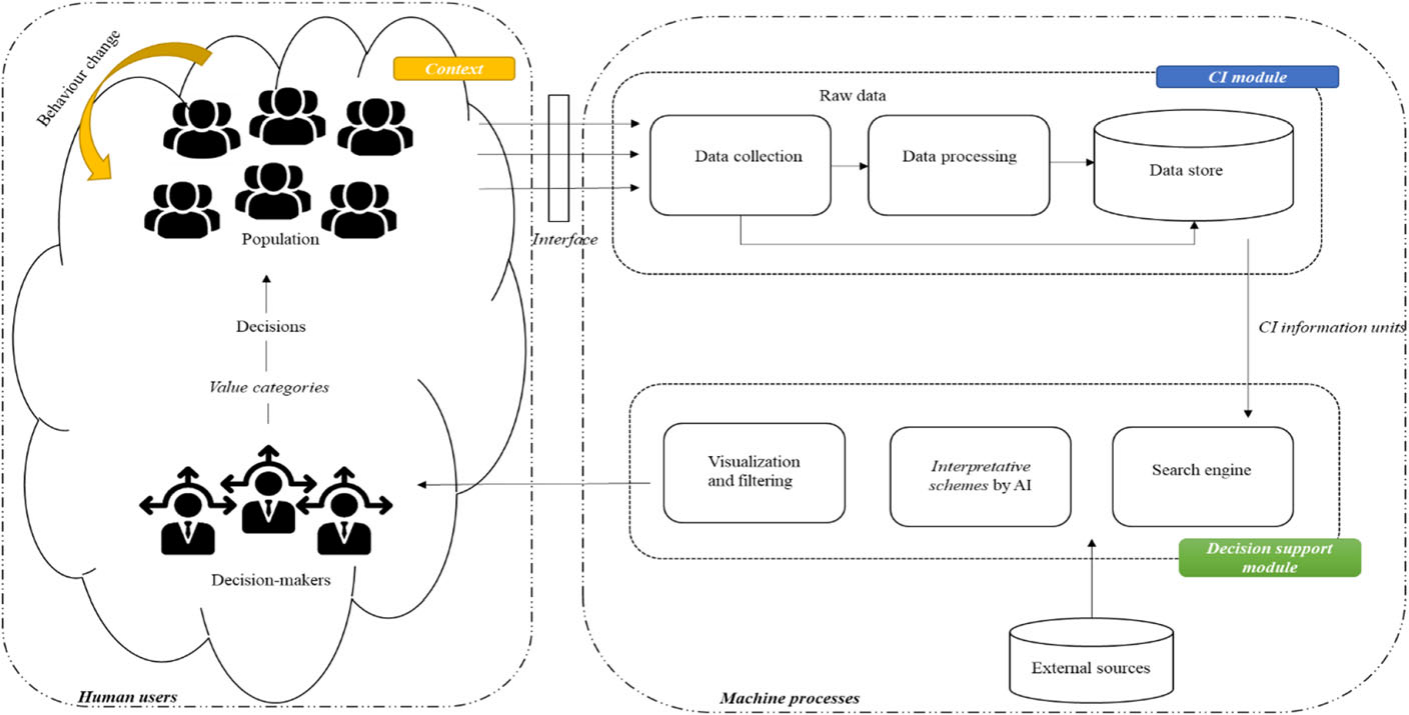
A viable framework based on collective knowledge in complex context. Source: Authors’ elaboration

Thanks to this model it is possible to depict an open and dynamic view of environmental dynamism as a source of opportunity in which the attention is on the relationships among multiple entities with the aim to define new forms of interaction (Saviano and Caputo 2013). Basically, the model represented in following Fig. 2 offers the opportunities for listing a few fundamental steps for supporting decision-making processes:

- Individuals produce data related to their perceptions and expectations using instruments provided by World Wide Web (Lastowka 2007);
- *CI module* collects and stores raw data from individuals’ actions defining semi-structured data which can be managed in a repository (Vivacqua and Borges 2012; Fan and Bifet 2013). In this phase, *information units* are produced and made available for multiple decision-making processes.
- *Decision Support module* receives semi-structured data and it processes them via AI algorithms for identifying useful data related to a specific decision-makers request (Gruber 2008). In this phase, machine learning algorithms via an AI approach are able to identify non-obvious and hidden patterns of information in data contributing to the definition of new *interpretative schemes*.
- Decision-makers analyse available structured data on the base of their tacit knowledge (Nonaka and Takeuchi 1995) and *value categories* (Barile 2009) for producing predictive knowledge thanks to analytics (Pauleen and Wang 2017). In this phase, value categories influence decision-makers in evaluating, revising, and accepting or rejecting contents emerged from data analysis (Hair 2007).
- Decisions are formulated on the basis of the decision-maker’s cognitive processes. As a consequence, each decision-maker can formulate different decisions. The implemented decisions, synthesis of subjective perceptions, and representations of available data (Ratten 2016; Carrubbo et al. 2017) entail a change in the behaviour of each individual of the population

In nutshell, the framework shown in Fig. 2 offers the opportunity for tracing renovate approaches to knowledge management thanks to the support provided by ICTs. The above-mentioned steps support dynamic interactions among the multiple actors involved in a shared and collaborate knowledge processes through which subjective contributions can be combined in order to obtain updated, useful, and integrate source of knowledge thanks to which it is possible to develop harmonic interactions for ensuring systems’ viability (Barile and Polese 2010; Caputo et al. 2017).

## Collective Intelligence for Facing COVID-19 Challenge: The Chinese Case

Reflections herein in previous sections could support both researchers and practitioners in better understanding the dynamics and challenges faced by the Chinese government in dealing with the pandemic caused by the COVID-19.

During the annual Lunar New Year holiday, COVID-19 has started to spread in China. In this period, the people returned to their family homes and several billion person-trips have made by residents and visitors, mostly on crowded planes, trains, and buses. Knowing this meant each infected person could have numerous close contacts over a protracted time and across long distances, the government needed to quickly act: China focused on traditional public health outbreak response tactics—isolation, quarantine, social distancing, and community containment (Wu and McGoogan 2020).

More than this, other approaches based on sophisticated computational methods have been applied by the Chinese government. Data from hundreds of millions of smartphones have been collected and used for containing COVID-19 spread. Data from all smartphones with enabled GPS have been collected for tracking the user’s itinerary and estimate the probability that an individual has exposure to COVID-19 by matching its position to the position of infected individuals or groups.

Thanks to these data, authorities have increased efficiency in the use of limited medical resources directing—for example—tests for the virus to high-risk subjects identified by the artificial intelligence algorithm and controlling individuals who may have attempted to flee quarantine (Goldman 2020).

Furthermore, Hu et al. (2020) developed a modified stacked auto-encoder for modelling the transmission dynamics of the epidemics. They used the latent variables in the auto-encoder and clustering algorithms to group the provinces/cities for investigating the transmission structure.

The aim of the work was to model the real-time forecasting of the confirmed cases of COVID-19 across China. The data were collected from January 11 to February 27, 2020, by World Health Organization. They predicted that the epidemics of COVID-19 will be over by the middle of April. If the data are reliable and there are no second transmissions, this model can forecast the transmission dynamics of the COVID-19 across the provinces/cities in China. The AI-inspired methods are a powerful tool for helping public health planning and policy-making.

In nutshell, big data and AI analytics have supported Chinese authorities in establishing the chain of virus transmission. On the 2nd of March, China reported only 126 new cases, compared to 851 in South Korea and 835 in Iran, out of a total of 1969 new worldwide cases (Goldman 2020) demonstrating the high accuracy of the AI-based methods for forecasting COVID-19 trajectory (Hu et al. 2020).

Anyways, all the approaches developed by Chines authorities have been made possible by Chinese value categories according to which no privacy constraints exist and telecom providers can collect and use locational data. Instead, smartphone users in the USA and Europe can access their own data, but privacy laws prevent the government from collecting these data.

Accordingly, it is possible to state that it is needed to increase data sharing in case of outbreaks or disasters for supporting global understanding and efficient decision-making processes (Allam and Jones 2020).

## Implications, Limitations, and Future Directions for Research

The increasing fluidity of information sharing made possible by the spread of World Wide Web has radically changed the world in which we all live every day (Sundararajan 2016; Constantiou et al. 2017; Caputo and Evangelista 2018). In such a scenario, social and business organizations are progressively understanding that the previous managerial models and business approaches, basically rooted on competitive advantages produced by the ownership of critical raw materials and/or efficiency in production, are not enough to ensure organizations’ survival.

New competitive arenas are emerging in which traditional rules seem to be not fully respected and different efforts are required to both organizations and individuals (Morgan and Hunt 1999; Hennig-Thurau and Hansen 2013). A new paradigm seems to be commonly recognized in all the emerging configurations: the central role of knowledge management processes supported by new technologies for better collecting, combining, and using data trough collaborative and participative approaches (Paroutis and Al Saleh 2009; Choudhary et al. 2013).

In such a scenario, the paper has provided preliminary reflections about the ways through which knowledge management processes can change thanks to the support provided by new technologies. Adopting the interpretative lens provided by the VSA, the key role of big data and AI in supporting the shift toward collective intelligence has been depicted and a possible framework has been drawn for supporting both researchers and practitioners in catching risks and opportunities of new technologies in knowledge management processes. In such a direction, the case of Chinese governmental approach to COVID-19 spread has been used as an example for clarifying the relevant role that information sharing and data accessibility have in ensuring efficient and fast decision-making approaches for facing disaster events.

The COVID-19 pandemic has underlined the multiple relevant advantages that a planned approach to collective knowledge management can provide in ensuring more efficiency in the management of unpredictable dynamics (Zhong et al. 2020) thanks to the support provided by new technologies (Hollander and Carr 2020). Investigated scenario recalls the attention on the need for improving shared approach to smart technologies in healthcare sector (Papa et al. 2020) with the aim to increase individual and collective ambidexterity through the definition of widen conceptual framework (Chinnaswamy et al. 2019) and the adoption of practicable instruments (Bresciani et al. 2018).

Reflections and pathway herein for supporting a shifting toward collective intelligence in knowledge management processes represent a starting point on which act for developing new managerial models able to stimulate data sharing and emphasise the role of new technologies for building new interpretative schemes able to overcome consolidated—and sometimes useless—representation of available data. From a different perspective, the paper also underlines the need for developing multidisciplinary research approaches able to overcome reductionist representations of business and social organizations. In such a direction, thanks to the interpretative contributions provided by systems thinking, the need for overcoming organizations’ boundaries in knowledge management practices is underlined and possible domains are underlined in which stimulate multidisciplinary debates for ensuring the emergence of collaborative and participative approaches trough which ensure both individual and collective satisfaction.

At the state, the paper enriches the ongoing debate about knowledge management processes in digital era provided theoretical reflections that require to be further developed through case studies and quantitative analysis able to provide information about advantages and risks of collective intelligence in different cognitive scenarios. Despite this, the paper highlights a possible key role of new technologies in supporting decision-making processes without forgetting and underestimating the relevant role of human resources.
